# Systematic Multiomic Analysis of *PKHD1L1* Gene Expression and Its Role as a Predicting Biomarker for Immune Cell Infiltration in Skin Cutaneous Melanoma and Lung Adenocarcinoma

**DOI:** 10.3390/ijms25010359

**Published:** 2023-12-26

**Authors:** Ji Young Kang, Jisun Yang, Haeryung Lee, Soochul Park, Minchan Gil, Kyung Eun Kim

**Affiliations:** 1 Department of Health Industry, Sookmyung Women’s University, Seoul 04310, Republic of Korea; ellykang@sookmyung.ac.kr (J.Y.K.); minchangil@gmail.com (M.G.); 2 Department of Cosmetic Sciences, Sookmyung Women’s University, Seoul 04310, Republic of Korea; jas11070@naver.com; 3 Department of Biological Sciences, Sookmyung Women’s University, Seoul 04310, Republic of Korea; frentoby@sookmyung.ac.kr (H.L.); scpark@sookmyung.ac.kr (S.P.)

**Keywords:** multiomic analysis, polycystic kidney and hepatic disease 1-like protein 1(PKHD1L1), immune infiltration, skin cutaneous melanoma (SKCM), lung adenocarcinoma (LUAD)

## Abstract

The identification of genetic factors that regulate the cancer immune microenvironment is important for understanding the mechanism of tumor progression and establishing an effective treatment strategy. Polycystic kidney and hepatic disease 1-like protein 1 (PKHD1L1) is a large transmembrane protein that is highly expressed in immune cells; however, its association with tumor progression remains unclear. Here, we systematically analyzed the clinical relevance of *PKHD1L1* in the tumor microenvironment in multiple cancer types using various bioinformatic tools. We found that the *PKHD1L1* mRNA expression levels were significantly lower in skin cutaneous melanoma (SKCM) and lung adenocarcinoma (LUAD) than in normal tissues. The decreased expression of *PKHD1L1* was significantly associated with unfavorable overall survival (OS) in SKCM and LUAD. Additionally, *PKHD1L1* expression was positively correlated with the levels of infiltrating B cells, cluster of differentiation (CD)-8^+^ T cells, and natural killer (NK) cells, suggesting that the infiltration of immune cells could be associated with a good prognosis due to increased *PKHD1L1* expression. Gene ontology (GO) analysis also revealed the relationship between *PKHD1L1*-co-altered genes and the activation of lymphocytes, including B and T cells. Collectively, this study shows that *PKHD1L1* expression is positively correlated with a good prognosis via the induction of immune infiltration, suggesting that *PKHD1L1* has potential prognostic value in SKCM and LUAD.

## 1. Introduction

Despite recent improvements in patient survival, cancer remains one of the leading causes of premature death in all countries of the world [1]. In particular, lung cancer is the leading cause of cancer death in both men and women, with an estimated 1.8 million deaths worldwide in 2020 [2]. In the United States, lung cancer caused approximately 135,000 deaths in 2020, accounting for nearly 25% of all cancer-related deaths [3]. Melanoma, the most aggressive skin cancer, accounts for only 1.7% of all skin cancers; however, it is responsible for 80% of skin cancer-related mortality [4,5]. In recent years, the understanding of cancer biology and new treatments, such as targeted therapy and immunotherapy, have helped reduce mortality; however, the mortality rates for melanoma and lung cancer remain high [5,6].

Oncogenesis is a complex process that includes the accumulation of multiple genetic alterations that cause uncontrolled cell growth, aberrant functions, and the modulation of the tumor microenvironment (TME) [7]. Various immune cells, of which the majority of the TME is comprised, are found in tumor tissues. Tumor-infiltrating lymphocytes (TILs) are tightly associated with the fate, invasiveness, and metastatic ability of tumors, as well as with the clinical outcomes in patients with cancer [8,9]. Therefore, the amount and composition of the TILs in tumor tissues are known to be associated with the prognoses of various malignant tumors [10,11,12]. Indeed, high levels of lymphocyte infiltration in melanoma patients are known to be associated with better prognoses. The presence of CD4^+^ and CD8^+^ lymphocytes at the tumor site in melanoma and non-small cell lung cancer has been reported to have good prognostic significance for the OS [13,14,15,16,17]. In particular, tumor-infiltrating B lymphocytes (TIBs) participate in both humoral and cellular immunity and have been shown to play opposing roles in anti-tumor activity and immunosuppressive subtypes [18,19,20]. Therefore, it is necessary to study the TIL population and its role in predicting the potential clinical prognosis.

Recently, various immunotherapies have been developed for the clinical treatment of cancer. Immunotherapy, which is based on the interaction between tumor cells and immune cells, has been effective in only a minority of cancer patients, and its mechanism is still unknown [21,22,23,24]. Because biomarkers can predict the advantages of a patient’s treatment strategy, they can be suggested as alternatives to overcome the selective therapeutic effect of immunotherapy [6].

Polycystic kidney and hepatic disease 1-like protein 1 (PKHD1L1) encodes fibrocystin-L, a transmembrane protein that includes a large extracellular domain (4190 amino acids (AAs)), transmembrane domain (21 AAs), and very small intracellular C-terminal domain (12 AAs) (https://www.uniprot.org/uniprot/Q86WI1: accessed on 15 January 2023) [25]. Its extracellular domain includes 13 Ig-like plexin transcription factor (IPT) domains, 2 G8 domains, and 9 parallel beta helix repeats (PbH1) [26]. These frequently appear in the extracellular domain of membrane proteins and are involved in interactions with possible ligands, such as polysaccharides [27,28,29]. Recent studies have reported an association between *PKHD1L1* expression, tumor progression, and genetic modifications. *PKHD1L1* is one of the most highly mutated genes in triple-negative breast cancer [30]. The *PKHD1L1* expression in thyroid cancer is downregulated compared to that in normal tissues. This downregulation is associated with various clinicopathological features, including the tumor volume, metastasis, the clinical stage, and lymph node metastasis [31,32]. However, the role of the *PKHD1L1* gene in various cancers has not yet been discussed. The aim of this study was to investigate the *PKHD1L1* expressions in various cancers using multiomic analysis, and to confirm the clinical relevance of *PKHD1L1* through the association between *PKHD1L1* expression and the survival of melanoma and lung cancer patients.

We comprehensively analyzed various publicly available expression datasets to determine the *PKHD1L1* expression levels and their prognostic values. The Gene Expression Profiling Interactive Analysis 2 (GEPIA2) database and Gene Expression database of Normal and Tumor Tissues 2 (GENT2) were used to confirm the mRNA expression of *PKHD1L1* in various types of tumors. The Tumor Immune Estimation Resource (TIMER) and TISIDB were utilized to determine the potential correlation between the *PKHD1L1* expression levels and TILs. Additionally, co-expression analysis and gene ontology (GO) were used to investigate the potential pathways associated with *PKHD1L1* in SKCM and LUAD. Collectively, our findings revealed that the *PKHD1L1* expression in SKCM and LUAD is positively correlated with patient survival and the infiltration of immune cells in tumors, including B cells, cluster of differentiation (CD)-8^+^ T cells, and natural killer (NK) cells. Therefore, we suggest *PKHD1L1* and its associated pathways as potential therapeutic targets for SKCM and LUAD.

## 2. Results

### 2.1. PKHD1L1 mRNA Expression Levels in Various Cancer Types

**Figure 1:**
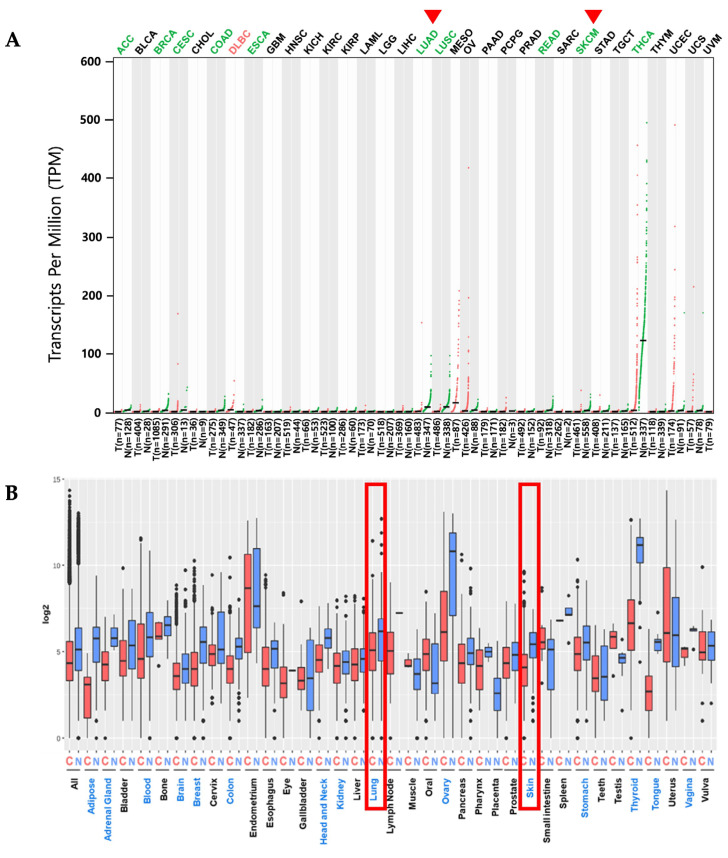
Polycystic kidney and hepatic disease 1-like protein 1 (*PKHD1L1*) mRNA expression levels in different types of cancers and their normal tissues. (**A**) *PKHD1L1* mRNA expression profiles across various tumor tissues and paired normal tissues were determined using the Gene Expression Profiling Interactive Analysis 2 (GEPIA2) database (https://gepia2.cancer-pku.cn: accessed on 25 April 2023). The green-dot plots in the graph represent the normal samples and the red-dot plots indicate the tumor samples. LUAD: T: *n* = 483; N: *n* = 347; SKCM: T: *n* = 461; N: *n* = 558. In GEPIA 2.0, the gene expression levels are expressed as log2 (TPM+1) (TPM: transcripts per million). The *PKHD1L1* expression in 33 cancer types was assessed via the log2-fold change. The *p*-value cutoff was 0.05, and the log2-fold change cutoff was 1. Red arrows represent the target cancers (LUAD, SKCM). Abbreviations of the names of various cancer types are listed in Supplementary Table S1. (**B**) *PKHD1L1* mRNA expression levels in various types of cancers were obtained from the Gene Expression database of Normal and Tumor Tissues 2 (GENT2) (https://medical-genomics.krib.re.kr/GENT2/: accessed on 25 April 2023). In the gene expression boxplot, the red plots represent the tumor tissues and the blue plots represent the normal tissues. Gene expression profiles of tumor samples and normal tissues of the target cancers (LUAD, SKCM) are shown in red frames. For statistics, this tool used two-sample *t*-tests. A *p*-value of <0.05 was considered statistically significant. Detailed statistical tests are shown in Supplementary Table S2. T: tumor N: normal.

We analyzed the differential expression patterns of *PKHD1L1* in various cancer types using web-based gene expression analysis tools, such as GEPIA2 and the GENT2. GEPIA2 was used to identify the *PKHD1L1* mRNA expressions in various cancers compared to normal tissues in RNA-sequencing data based on TCGA and GTEx datasets. The *PKHD1L1* mRNA expression was significantly downregulated in multiple cancers, including adrenocortical carcinoma (ACC), breast invasive carcinoma (BRCA), cervical squamous cell carcinoma and endocervical adenocarcinoma (CESC), esophageal carcinoma (ESCA), LUAD, lung squamous cell carcinoma (LUSC), rectum adenocarcinoma (READ), SKCM, and thyroid carcinoma (THCA), compared with normal tissues (Figure 1A). We used the GENT2 to analyze the expression levels of multiple cancers from the microarray-based gene expression datasets profiled by the Affymetrix u133 platform. Figure 1B shows that the *PKHD1L1* mRNA expression was downregulated in 14 cancer types, including skin and lung cancer. Increased *PKHD1L1* expression was observed only in lymphoid neoplasm diffuse large B cell lymphoma (DLBCL) in the GEPIA. Collectively, the results of this study showed that the mRNA expression of the *PKHD1L1* gene in several types of cancer, including SKCM and LUAD, was significantly lower than that in normal tissues.

### 2.2. Prognostic Values of PKHD1L1 mRNA Expression Levels in Various Types of Cancers

**Figure 2:**
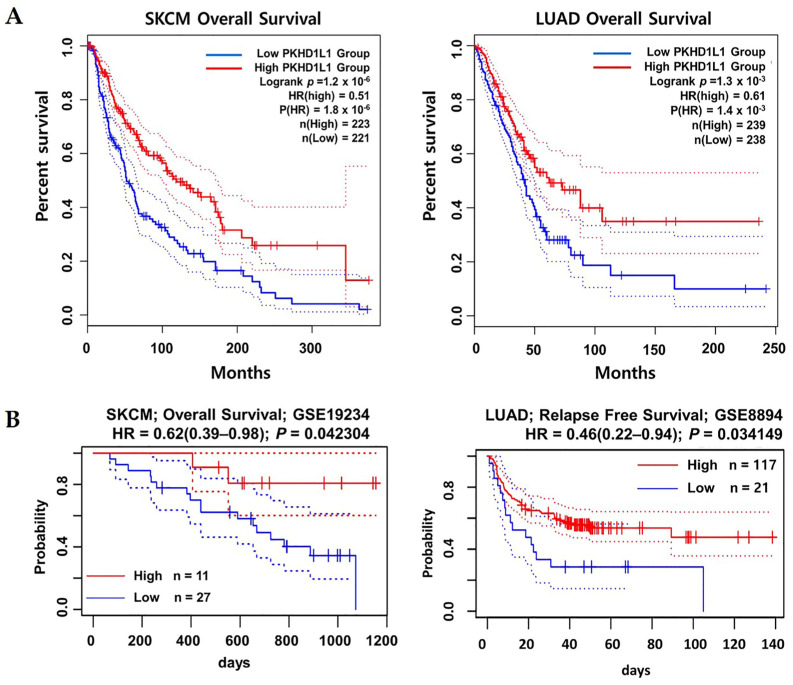
Correlation between *PKHD1L1* expression levels and prognoses of skin cutaneous melanoma (SKCM) and lung adenocarcinoma (LUAD). (**A**) Kaplan–Meier plots of the relationship between the *PKHD1L1* gene expression levels and survival rates of patients with SKCM and LUAD. The survival curves were used to determine the survival rates of the patients with high (red) and low (blue) *PKHD1L1* expression levels in SKCM and LUAD using the GEPIA2 web tool (http://gepia2.cancer-pku.cn/: accessed on 25 April 2023). (**B**) Kaplan–Meier survival curves retrieved from PrognoScan (http://www.prognoscan.org/: accessed on 27 April 2023) for *PKHD1L1* mRNA expression levels with the SKCM dataset GSE 19234 and LUAD dataset GSE 8894. The log-rank *p*-value, *p*-value of the HR, Cox proportional hazard ratio (HR), and number of patient groups are included. The 95% confidence intervals are shown with dotted lines.

To explore the association between the *PKHD1L1* mRNA expression and patient prognoses in various cancers, the OS rates were compared between two patient groups with high and low *PKHD1L1* expressions in various TCGA datasets using the OncoLnc online tool (http://www.oncolnc.org/: accessed on 3 May 2023). The Cox regression results for the *PKHD1L1* expressions in various cancers are presented in Supplementary Table S3. Five cancer types, SKCM, pancreatic adenocarcinoma (PAAD), LUAD, liver hepatocellular carcinoma (LIHC), and stomach adenocarcinoma (STAD), showed significant correlations between the overall patient survival and *PKHD1L1* mRNA expression (*p* < 0.01). Among the five cancer types, SKCM and LUAD showed the highest correlations and decreased *PKHD1L1* expressions compared to their normal counterparts (Figure 1). Therefore, we selected SKCM and LUAD for further analyses. Kaplan–Meier survival curves with TCGA datasets were retrieved from the GEPIA2 database for SKCM, LUAD, and other cancer types. The most significant positive correlations between the *PKHD1L1* mRNA expression levels and patient survival rates were found in SKCM (log-rank *p* = 1.2 × 10^−6^) and LUAD (log-rank *p* = 1.3 × 10^−3^), while negative correlations were observed in kidney renal papillary cell carcinoma (KIRP), PAAD, and STAD (Figure 2, Supplementary Figure S2). Additionally, we performed a survival analysis in skin and lung cancer datasets using the PrognoScan database to investigate the prognostic implications of *PKHD1L1* expression. The results also showed that *PKHD1L1* expression was positively associated with patient survival in the melanoma dataset GSE19234 (OS, hazard ratio (HR) = 0.62 (0.39–0.98), *p* = 0.042304) and lung cancer dataset GSE8894 (relapse-free survival (RFS), HR = 0.46 (0.22–0.94), *p* = 0.034149) (Figure 2B). Moreover, the association between the *PKHD1L1* expression and OS in the clinicopathological parameters, including sex, age, and tumor stage, was analyzed in the SKCM-TCGA and LUAD-TCGA datasets using the R2 web tool (Supplementary Figure S1). Significantly better patient survival in the high-*PKHD1L1*-expression group was found in the 30-years-and-older patient age groups in SKCM and in the 50-years-and-older groups in LUAD. A positive correlation between patient survival and *PKHD1L1* expression was also observed in cancer stages 1, 2, and 3 in SKCM and LUAD, but not in stage 4 in SKCM. Overall, *PKHD1L1* expression was remarkedly positively associated with patient survival in SKCM and LUAD, suggesting its prognostic value in SKCM and LUAD.

### 2.3. Correlation of PKHD1L1 Expression and Immune Cell Infiltration

**Figure 3:**
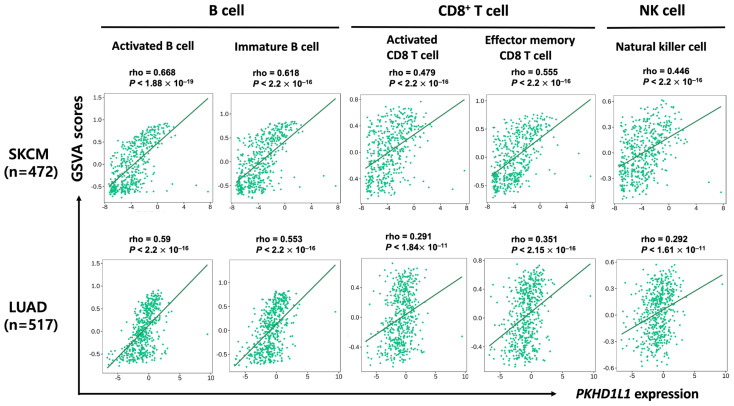
Correlation between *PKHD1L1* expression and immune cell infiltration in SKCM and LUAD. The correlation of the *PKHD1L1* expression with TILs in SKCM and LUAD was investigated using the TISIDB web tool (http://cis.hku.hk/TISIDB/index.php/: accessed on 13 May 2023). The *PKHD1L1* expression had significant positive correlations with activated B cells (rho = 0.668), immature B cells (rho = 0.618), activated CD8^+^ T cells (rho = 0.479), effector memory CD8^+^ T cells (rho = 0.555), and NK cells (rho = 0.446) in SKCM. The *PKHD1L1* expression had positive correlations with activated B cells (rho = 0.59), immature B cells (rho = 0.553), activated CD8^+^ T cells (rho = 0.291), effector memory CD8^+^ T cells (rho = 0.351), and NK cells (rho = 0.292) in LUAD.

Because SKCM and LUAD are hot tumors with high lymphocyte contents and responsiveness to immune checkpoint inhibitors [33,34], we analyzed the association between the *PKHD1L1* expression and infiltrated immune cell types. For this, we used the TIMER version 1.0 database (https://cistrome.shinyapps.io/timer/: accessed on 3 May 2023). The results showed that the *PKHD1L1* expression was significantly correlated with B cells and CD8^+^ T cells in SKCM and LUAD (Table 1). We further analyzed whether the *PKHD1L1* expression levels were correlated with the specific immune cell infiltration in SKCM using the TISIDB web tool. The *PKHD1L1* expression was significantly positively correlated with the infiltrating levels of activated B cells (r = 0.668, *p* < 2.2 × 10^−16^), immature B cells (r = 0.618, *p* < 2.2 × 10^−16^), activated CD8^+^ T cells (r = 0.479, *p* < 2.2 × 10^−16^), effector memory CD8^+^ T cells (r = 0.555, *p* < 2.2 × 10^−16^), and NK cells (r = 0.446, *p* < 2.2 × 10^−16^). In LUAD, the *PKHD1L1* expression was also positively correlated with the infiltration levels of activated B cells (r = 0.59, *p* < 2.2 × 10^−16^), immature B cells (r = 0.553, *p* < 2.2 × 10^−16^), activated CD8^+^ T cells (r = 0.291, *p* < 1.84 × 10^−11^), effector memory CD8^+^ T cells (r = 0.351, *p* < 2.15 × 10^−16^), and NK cells (r = 0.292, *p* < 1.61 × 10^−11^) (Figure 3). These results demonstrate a positive correlation between *PKHD1L1* expression and the immune cell infiltration levels of B cells, CD8^+^ T cells, and NK cells.

Additionally, to further examine the immune infiltration estimations, we utilized the EPIC, QUANTISEQ, MCP-COUNTER, CIBERSORT, CIBERSORT-ABS, and XCELL algorithms with the TIMER version 2.0 database (http://timer.cistrome.org/: accessed on 3 May 2023). We analyzed the correlation between the *PKHD1L1* expression and the immune infiltration levels of B cells, CD8^+^ T cells, and NK cells after adjusting for the tumor purity. As shown in Table 2, *PKHD1L1* was positively correlated with B cells, CD8^+^ T cells, and NK cells in both SKCM (MCP_COUNTER; B cells (rho = 0.542, *p* = 2.56 × 10^−36^), CD8^+^ T cells (rho = 0.433, *p* = 7.25 × 10^−72^), NK cells (rho = 0.433, *p* = 2.40 × 10^−22^)) and LUAD (MCP_COUNTER; B cells (rho = 0.551, *p* = 2.08 × 10^−40^), CD8^+^ T cells (rho = 0.279, *p* = 2.73 × 10^−10^), NK cells (rho = 0.263, *p* = 1.47 × 10^−10^)). Interestingly, *PKHD1L1* was positively correlated with the infiltration of activated NK cells (CIBERSORT_ABS; SKCM (rho = 0.326, *p* = 9.09 × 10^−13^) and LUAD (rho = 0.22, *p* = 8.399 × 10^−7^)); however, it had a negative correlation with that of resting NK cells (CIBERSORT_ABS; SKCM (rho = −0.164, *p* = 4.45 × 10^−4^) and LUAD (rho = −0.05, *p* = 2.68 × 10^−1^)). Taken together, these results suggest that *PKHD1L1* expression is highly correlated with the abundance of infiltrated B, CD8^+^ T, and NK cells, which may repress cancer progression in SKCM and LUAD.

### 2.4. Expression of PKHD1L1 in Various Types of Immune Cells

**Figure 4:**
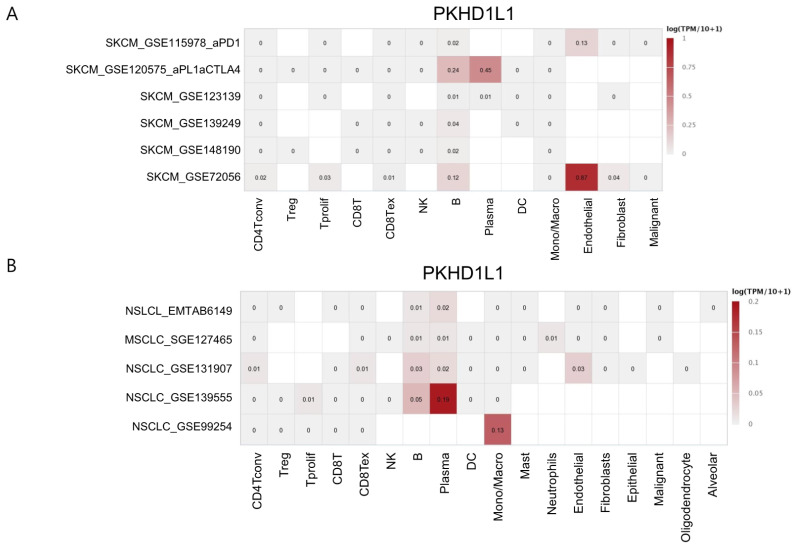
Single-cell RNA-sequencing analysis of *PKHD1L1* in various cell types in (**A**) melanoma and (**B**) lung cancer. Number in each box indicates the TPM of *PKHD1L1* transcript from single-cell sequencing data.

The strong correlation between the *PKHD1L1* expression and the number of infiltrated B cells, CD8^+^ T cells, and NK cells suggests that the immune cells in the tumor tissue could express *PKHD1L1.* We investigated the expression levels of various immune cell types using the DICE database, which provides data on the immune cell gene expression in healthy individuals. As shown in Supplementary Figure S3, the expression levels of *PKHD1L1* are exclusively high in naive B cells. We analyzed the *PKHD1L1* expression in single-cell RNA-sequencing datasets using the TISCH database to determine the cell types expressing *PKHD1L1* in melanoma and NSCLC. As shown in Figure 4, the *PKHD1L1* expression was the highest in B cells and plasma cells in most datasets, suggesting that the *PKHD1L1* expression is high in these infiltrated cells in melanoma and NSCLC. *PKHD1L1* expression was also found in other cell types, including T cells, fibroblasts, and endothelial cells, in some datasets. Additionally, the *PKHD1L1* expression was found to be predominantly high in B cells and plasma cells in GSE120575, in which samples were collected from melanoma patients treated with checkpoint inhibitors, anti-programmed death receptor 1 (PD-1), and anti-cytotoxic T-lymphocyte-associated protein-4 (CTLA-4) [35], as shown in Figure 4. Melanoma is highly reactive to checkpoint inhibitors, and CTLA-4 and PD-1 are representative immune checkpoint molecules [36]. Although these molecules target T-cell functions, it has been reported that the maturation and differentiation of B cells are positively correlated with reactivity to these checkpoint inhibitors [37]. Therefore, we propose that the expression of *PKHD1L1* in melanoma has a high prognostic value for predicting effective therapeutic outcomes.

### 2.5. Analysis of Genes Co-Expressed with PKHD1L1

**Figure 5:**
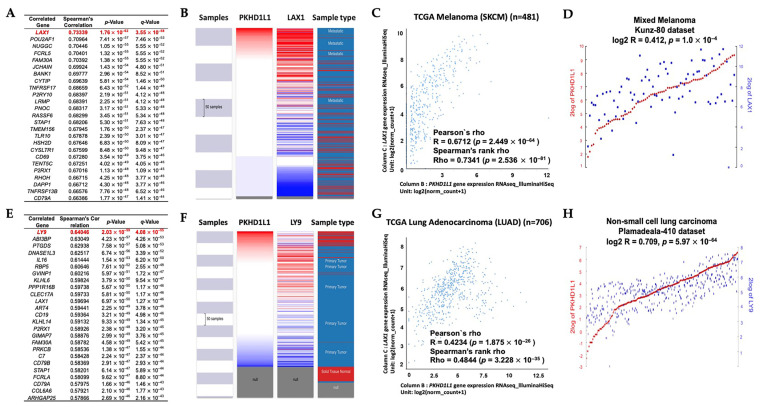
Profiling of co-expressed genes with *PKHD1L1*. Co-expression profile of the *PKHD1L1* gene in SKCM and LUAD. (**A**) The top 25 genes positively associated with the *PKHD1L1* transcript level based on TCGA with SKCM. (**B**) A heatmap revealed the *PKHD1L1* and *LAX1* mRNA expression levels using the UCSC Xena Browser (https://xena.ucsc.edu/: accessed on 17 July 2023). (**C**) Dot plot of *PKHD1L1* and *LAX1* mRNA expressions in the TCGA-SKCM dataset. (**D**) The correlation module showed the expression scatterplots between *PKHD1L1* mRNA and *LAX1* in the Mixed Melanoma-Kunz-80 dataset via the R2 platform (https://hgserver1.amc.nl/cgi-bin/r2/main.cgi: accessed on 28 July 2023). (**E**) The *LY9* genes with the highest positively correlation with *PKHD1L1* transcript level in LUAD. (**F**) A heatmap revealed the *PKHD1L1* and *LY9* mRNA expression levels. (**G**) Dot plot of *PKHD1L1* and *LY9* mRNA expressions in the TCGA-LUAD dataset. (**H**) Correlation between *LY9* and *PKHD1L1* mRNA expressions in the tumor non-small cell lung carcinoma-Plamadeala-410 dataset, as determined using the R2 database.

Next, we investigated genes co-expressed with *PKHD1L1* using the cBioPortal database (https://www.cbioportal.org/: accessed on 23 May 2023) in the TCGA-SKCM and TCGA-LUAD datasets. Among these genes, the expression of lymphocyte transmembrane adaptor 1 (*LAX1*) was the most highly co-expressed in SKCM (R = 0.733, *p* = 1.0 × 10^−62^) (Figure 5A). We also confirmed the positive correlation between *PKHD1L1* and *LAX1* using Pearson (r = 0.6712, *p* = 2.449 × 10^−63^) and Spearman (rho = 0.7341, *p* = 2.536 × 10^−81^) correlation analyses with TCGA data from SKCM patients using the UCSC Xena web tool (Figure 5B,C). A strong association between the expressions of *PKHD1L1* and *LAX1* was also confirmed using the Mixed Melanoma-Kunz-80 dataset with the R2 database (R = 0.412, *p* = 1.0 × 10^−4^) (Figure 5D). Additionally, *LY9* was the most highly co-expressed in LUAD (R = 0.640, *p* = 2.0 × 10^−59^) (Figure 5E). Figure 5F,G shows that the strong association between the *PKHD1L1* and *LY9* expressions was also confirmed using Pearson (r = 0.4234, *p* = 1.975 ×10^−26^) and Spearman (rho = 0.4844, *p* = 3.228 × 10^−35^) correlation analyses in the TCGA-LUAD dataset. In addition, the expressions of *PKHD1L1* and *LY9* were positively correlated in the NSCLC-Plamadeala-410 dataset (R = 0.709, *p* = 5.97 × 10^−64^) (Figure 5H). Our findings suggest that the expression of *PKHD1L1* and those of highly co-expressed genes in SKCM and LUAD might be closely correlated.

### 2.6. Prediction of Role of PKHD1L1 Using GeneMANIA and Ontology and Its Co-Expressed Genes

**Figure 6:**
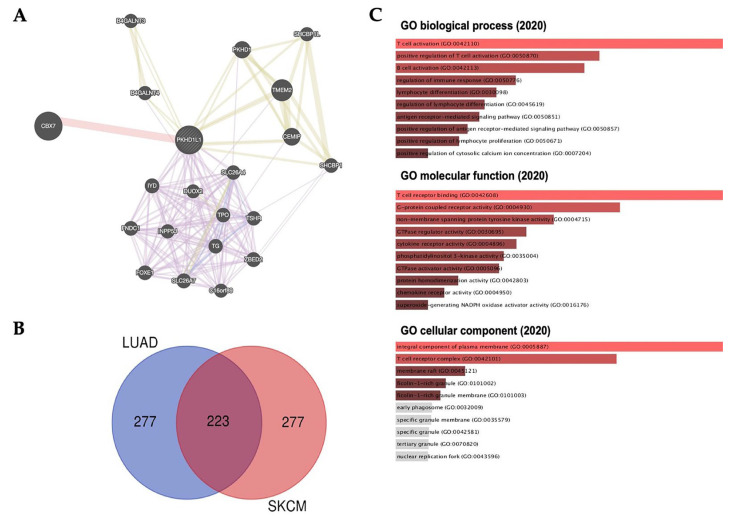
Cluster and gene ontology (GO) analysis of the *PKHD1L1* gene in SKCM and LUAD. (**A**) Protein–protein interaction (PPI) network of *PKHD1L1* using GeneMANIA (https://genemania.org/: accessed on 13 August 2023). PPI network analysis revealed the gene sets that interact closely with *PKHD1L1*. Various networks, such as co-expressions, pathways, physical or genetic interactions, and shared protein domains, are shown in different colors. (**B**) The Venn Diagram was used for the number of specific common genes between SKCM (*n* = 277) and LUAD (*n* = 277) correlated with *PKHD1L1* (*n* = 223). (**C**) Co-expressed gene profiles with the *PKHD1L1* gene involved in signaling pathways between SKCM and LUAD. Gene ontology (GO) analysis was performed using the *PKHD1L1* gene and its 223 common co-expressed genes via the Enrichr web tool (https://amp.pharm.mssm.edu/Enrichr: accessed on 15 August 2023) for GO biological processes (2020), molecular functions (2020), and cellular components (2020).

To predict the function of *PKHD1L1*, we performed a cluster analysis of the interactions involving *PKHD1L1* using the GeneMANIA web tool. The predicted protein partners of PKHD1L1 were CBX7 of the physical interactions, TMEM2, CEMIP, PKHD1, SHCBP1, SHCBP1L, and CEMIP of the shared protein domains, and SLC26A7, ZBED2, FOXE1, FNDC1, TG, SHCBP1, TPO, SLC26A4, TSHR, B4GALNT3, B4GALNT4, INPP5J, SHCBP1L, C16orf89, and DUOX2 (Figure 6A). Furthermore, we carried out an ontology analysis of the commonly correlated genes in SKCM and LUAD to identify their potential biological relevance. We identified 223 overlapping genes from the 500 most highly positively correlated genes in SKCM and LUAD by using a Venn diagram (Figure 6B, listed in Supplementary Table S4).

Next, GO analysis was carried out with *PKHD1L1* and its positively related genes for biological processes, molecular functions, and cellular components using the Enrichr database. *PKHD1L1* and 233 co-altered genes were mainly associated with T- or B-cell activation in the GO biological processes, T-cell receptor binding in the GO molecular functions, and integral components of the plasma membrane in the GO cellular components (Figure 6C). Taken together, the gene enrichment analysis of *PKHD1L1* and its co-altered gene set showed that *PKHD1L1* could be associated with the lymphocyte activation signaling pathways. Similar to our results, a previous study reported that *PKHD1L1* is underexpressed in thyroid cancer and may act as a tumor suppressor gene in the progression of thyroid cancer. However, interestingly, there was no significant relationship between the *PKHD1L1* expression and survival in thyroid cancer (Supplementary Table S3).

## 3. Discussion

PKHD1L1, an extracellular protein with a single transmembrane domain, is known to play an important role in cellular immunity. It is known that *PKHD1L1* shows low-level expression in many primary immune cell subtypes; however, its expression tends to be specifically upregulated in activated T cells [32]. In addition, *PKHD1L1* transcripts have been detected in organs fully composed of immune cell subtypes, such as the spleen and thymus, as well as activated T cells and B cells [32]. This suggests that *PKHD1L1* plays an important role in lymphocyte activation and provides crucial functions in the cellular immunity against cancer cells. Recently, *PKHD1L1* was found to be a tumor suppressor associated with thyroid cancer cell progression; however, the clinical relevance of the *PKHD1L1* expression in various types of cancers has not yet been studied [31]. Therefore, we systematically analyzed the expression of *PKHD1L1* from publicly available cancer expression datasets and determined the relevance of the *PKHD1L1* expression and patient survival rates in hot tumors. As shown in Figure 1 and Figure 2 and Supplementary Table S3, *PKHD1L1* was lower in most tumor tissues than in normal tissues, and positively correlated with survival rates in SKCM and LUAD, suggesting that *PKHD1L1* expression may be a favorable prognostic factor. Similar to our results, a previous study reported that *PKHD1L1* is underexpressed in thyroid cancer and may act as a tumor suppressor gene in the progression of thyroid cancer. However, interestingly, there was no significant relationship between the *PKHD1L1* expression and survival in thyroid cancer (Supplementary Table S3). Furthermore, our data showed a strong positive correlation between *PKHD1L1* expression and immune cell infiltration (Figure 3), suggesting that *PKHD1L1* expression has prognostic value through the infiltration of immune cells, such as B cells, CD8^+^ T cells, and NK cells, into the TMEs of SKCM and LUAD.

Recently, an increasing number of studies have elucidated the role of TILs as prognostic biomarkers for various cancers. TILs are a heterogeneous group of lymphocytes that enhance anti-tumor immune responses or suppress immune responses, and their presence tends to modulate robust immune responses in most TMEs [38,39]. The TME consists of a variety of immune cells, including T and B lymphocytes, NK cells, DCs, and macrophages, as well as a population of cancer cells in the tumor mass [40,41]. Importantly, the composition of immune cells in the TME is known to determine the tumor progression and clinical outcome, and thus immune cell infiltration into the TME is associated with good prognoses in various cancers, such as lung cancer and melanoma [9,42]. TILs are composed of various lymphocytes, including NK cells, CD4^+^ T cells, CD8^+^ T cells, B cells, macrophages, and DCs [43]. In particular, in this study, *PKHD1L1* was highly expressed in B cells in SKCM and LUAD, and the high positive correlation between the expression of *PKHD1L1* and the immune infiltration of B cells was confirmed (Figure 3). Many studies have reported that TIBs can be observed in various solid tumors and are correlated with good prognoses in colorectal cancer, hepatocellular carcinoma, pancreatic ductal adenocarcinoma, and head and neck squamous cell carcinoma [44,45,46]. TIBs are known to regulate antigen presentation, antibody production, and immune homeostasis, and to activate the immune response of T cells [47]. In TMEs, TIBs inhibit tumor progression by promoting T-cell responses and secreting immunoglobulins. In addition, they can directly kill cancer cells by exhibiting cytotoxic activity via the activation of the Fas/Fas L pathway and the secretion of cytolytic molecules, such as granzyme B (GZMB) and tumor necrosis factor-related apoptosis-inducing ligand (TRAIL) [48,49,50]. In fact, B-cell depletion using anti-CD20 antibody in a B16 melanoma murine model led to the development of pulmonary metastasis [51]. Furthermore, recent studies have shown that the activation of B-cell receptor (BCR) signaling could be associated with good prognoses in various cancers, such as breast cancer and lung cancer, by generating humoral immune responses via TIBs, resulting in effective anti-tumor immunity at the tumor site [52,53]. BCR signaling is essential for normal B-cell development and adaptive immunity. Activation mechanisms of BCR signaling include persistent BCR stimulation by various antigens present in TMEs, downstream signaling components, and ligand-independent BCR signaling [54]. The diversity of the BCR repertoire via BCR recombination is an especially favorable prognostic factor in SKCM and LUAD [55,56]. Collectively, we suggest that *PKHD1L1* expressed in SKCM and LUAD could be associated with the infiltration of B cells and the activation of TIBs via BCR signaling, resulting in good prognoses in both types of cancer.

In addition to the role of B cells in the TME, tumor-infiltrating CD8^+^ T cells and NK cells are associated with good clinical outcomes and play an important role in anti-tumor efficacy and survival [57,58,59,60]. Many studies have reported that activated NK and CD8^+^ T cells play important roles in melanoma and lung cancer [61,62,63]. Both activated CD8^+^ T cells and NK cells show cytolytic activity against tumor cells by secreting several cytolytic molecules, such as granzyme A (GZMA), GZMB, and perforin (PRF1), showing good prognoses in various types of cancers [61,64]. Our previous study also revealed that genes positively correlated with the infiltration of these effector cells were associated with a good prognosis in SKCM [43,65,66]. Although activated CD8^+^ T cells and NK cells play important roles in the TME, our data show that in SKCM, the *PKHD1L1* expression was the highest in B cells, with little expression in CD8^+^ T cells and NK cells (Figure 4 and Supplementary Figure S3). However, the *PKHD1L1* expression had a strong positive correlation with CD8^+^ T, NK, and B cells, as shown in Figure 3, implying that the infiltration of CD8^+^ T and NK cells may be associated with the expressions of other genes regulated by *PKHD1L1* in B cells. Moreover, CD4^+^ and CD8^+^ T cells activated by APCs induce the upregulation of *PKHD1L1*, and the *PKHD1L1* protein in activated T cells can be secreted or bound to the cell surface as a ligand [32]. The program resulting from the interaction between T cells and antigen-presenting cells (APCs) regulates immune functions, such as T-cell proliferation, cytokine production, and cytotoxicity, which are essential for immune surveillance against tumors [67,68,69]. Therefore, *PKHD1L1* may have the potential to play a role in regulating cytokine production and cytotoxicity for immune surveillance within the TME. GO analysis showed that chemokine receptors and activity were positively correlated with *PKHD1L1* expression (Figure 6). Therefore, to investigate how the increased expression of *PKHD1L1* affects the infiltration of CD8^+^ T cells and NK cells, we analyzed the correlation between the *PKHD1L1* expression and the chemokine genes associated with the infiltration of CD8^+^ T cells and NK cells into the TME. Supplementary Figure S4 shows that the expressions of several chemokines, such as chemokine (C-C motif) ligand 4 (CCL4), chemokine (C-C motif) ligand 5 (CCL5), chemokine (C-C motif) ligand 19 (CCL19), and chemokine (C-X-C) motif ligand 9 (CXCL9), which are known to induce the migration of CD8^+^ T cells and NK cells, have highly positive correlations with the *PKHD1L1* expression in both SKCM and LUAD. It is well known that the cytolytic activity of NK cells is augmented by CCL4 and CCL5, and CD8^+^ T cells have their corresponding chemokine receptors [70,71]. In addition, previous studies have shown that the expressions of CCL4 and CCL5 are positively correlated with the infiltration of NK cells and CD8^+^ T cells in melanoma, and the increased secretion of CCL5 recruits CD8^+^ T cells to the TME in LUAD [72,73]. In addition to CCL4 and CCL5, NK cells have the chemokine receptor CCR7, which is the receptor for the CCL19 mediation of T-cell recruitment [74,75]. Effector CD8^+^ T cells and NK cells express CXCR3 as a major receptor that drives their recruitment to the tumor site, and CXCL9 is a known ligand of CXCR3 [76]. In melanoma and lung cancer, CXCL9 is positively correlated with the infiltration of CD8^+^ T and NK cells for anti-tumor responses [72,77]. As shown in Supplementary Table S4, CCL19 and the chemokine receptors CCR7 and CXCR3 were co-expressed with *PKHD1L1* in both SKCM and LUAD. Collectively, the highly correlated expression of *PKHD1L1* and chemokines attracting CD8^+^ T cells and NK cells suggests that an increase in chemokines in *PKHD1L1*-high tumors could promote the infiltration of CD8^+^ T cells and NK cells into SKCM and LUAD tissues.

In the present study, we identified the biological processes associated with *PKHD1L1* and its co-altered genes in SKCM and LUAD. In SKCM, the *LAX1* expression was the most significantly co-altered with *PKHD1L1* expression (Figure 5A). LAX1 is associated with BCR signaling, which plays an important role in chronic lymphocytic leukemia pathogenesis, and its expression is highly correlated with B-cell infiltration in periodontitis [78,79]. BCRs are essential for the integration of various signals that induce immune responses and have anti-tumor effects through interactions with various immune cells in the TME. The binding of BCRs and tumor antigens not only increases the secretion of cytokines that induce the activation of CD4^+^ T cells, CD8^+^ T cells, and NK cells, but also directly kills tumor cells through the secretion of cytolytic molecules, such as GZMB and TRAIL [49]. Furthermore, LAX1 plays a role in T-cell activation by coupling the TCR ligation at the membrane to signaling cascades and associates with Grb2, Gads, and other signaling molecules [80]. In LUAD, *Ly9* was the most significantly co-altered with *PKHD1L1* expression (Figure 5E). Lymphocyte antigen 9 (Ly9), otherwise known as CD229, is a member of the signaling lymphocyte activation molecule (SLAM) family [81,82]. SLAM family receptors and SLAM-related protein (SAP) adapters regulate the development and function of T cells, cytokine production, and the main tissue complex-independent cell inhibition of NK cells by activating NK and T lymphocytes [83]. Additionally, as shown in Figure 5A and 5E, *FAM30A*, *STAP1*, *P2RX1*, and *CD79A* were also co-expressed with *PKHD1L1* in SKCM and LUAD. It has been reported that the expression of lncRNA FAM30A is high in B cells and correlates with the expressions of immunoglobulin genes, and the STAP1 protein is mainly expressed in immune tissues, including the thymus, spleen, lymph nodes, and bone marrow, and especially in B cells [84]. P2RX1 can interact closely with naive B cells in LUAD, and CD79a is a signaling component of the pre-B-cell receptor [85,86]. Furthermore, *PKHD1L1*-co-expressed genes were the most significantly enriched in immune cell activities, such as T-cell receptor binding, the positive regulation of T-cell activation, and B-cell activation. Collectively, our results suggest that *PKHD1L1* can enhance the activity of other immune cells through B-cell activation by regulating BCR signaling, especially in SKCM and LUAD, resulting in a good prognosis via effective anti-tumor mechanisms. However, as this study has limitations in predicting the clinical relevance using various multiomic techniques, additional in vitro and in vivo experiments are required to demonstrate the role of *PKHD1L1* in the activity and signal transduction of various immune cells.

## 4. Materials and Methods

### 4.1. Analysis of PKHD1L1 mRNA Expression Levels in Various Types of Cancers

The mRNA expression levels of *PKHD1L1* in various types of cancers and normal tissues were analyzed using Gene Expression Profiling Interactive Analysis 2 (GEPIA2) (http://gepia2.cancer-pku.cn/: accessed on 25 April 2023) and the Gene Expression database of Normal and Tumor Tissues 2 (GENT2) (http://gene2.appex.kr/gent2/: accessed on 25 April 2023) [87]. In GEPIA2, the *PKHD1L1* expressions in tumor samples from The Cancer Genome Atlas (TCGA) database were compared to the combined expression data of paired adjacent TCGA and Genotype Tissue Expression project (GTEx) normal tissues [87]. The GENT2 provides microarray-based gene expression profiles across normal tissues and various types of cancers in the Affymetrix U133plus2 of the GPL570 platform. All settings were set to default. The abbreviations for various types of cancer are listed in Supplementary Table S1.

### 4.2. Analysis of Relationship between PKHD1L1 Expression Levels and Patient Survival Rates

We first screened the types of cancers in which the *PKHD1L1* expression was correlated with patient survival using OncoLnc (http://www.oncolnc.org/: accessed on 3 May 2023) across TCGA datasets. The correlation between the *PKHD1L1* expression levels and survival rates was analyzed using the GEPIA2 and PrognoScan (http://dan00.bio.kyutech.ac.jp/prognoscan/: accessed on 27 April 2023) databases for skin and lung cancers [87]. Patient samples were split into two groups based on the median values of the *PKHD1L1* expression and analyzed using the log-rank test in GEPIA2 and Kaplan–Meier survival curves. PrognoScan is a database that includes prognostic data for various types of cancers [88]. The correlation between the gene expression and patient survival in the GSE19234 and GSE8894 datasets was estimated using PrognoScan with a Cox *p*-value < 0.05. OS analysis was performed using the SKCM-TCGA (*n* = 468) and LUAD-TCGA (*n* = 515) datasets for sex, age, and tumor stage using the R2 database (Supplementary Figure S1) (* *p* < 0.05).

### 4.3. Analysis of PKHD1L1 Expression Levels in Various Types of Immune Cells

The databases for immune cell expression, expression quantitative trait loci (eQTLs), and epigenomics (DICE) (https://dice-database.org/landing/: accessed on 15 May 2023) were used to determine the expression levels of *PKHD1L1* in various types of immune cells. DICE includes the transcriptomes of common human immune cell types generated via the RNA-sequencing (RNA-seq) analysis of samples from healthy subjects [89]. The number of transcripts per million (TPM) represents the gene expression level in each immune cell type. To examine the detailed expression levels of *PKHD1L1* in various tumor-associated cell types in melanoma and non-small cell lung cancer (NSCLC), we employed a tumor immune single-cell hub (TISCH) (http://tisch.comp-genomics.org/: accessed on 15 May 2023). We retrieved the expression characteristics of *PKHD1L1* from six melanoma datasets and five NSCLC datasets. TPM counts are presented for each type of immune cell for each dataset.

### 4.4. Analysis of Correlation of PKHD1L1 Expression Levels with Immune Cell Infiltration

The TIMER v.1.0 database (http://cistrome.shinyapps.io/timer/: accessed on 3 May 2023) was used to analyze the association between the *PKHD1L1* expression and immune infiltrates [90]. The correlation between the *PKHD1L1* expression levels and the enrichment of each type of immune cell infiltration in the tumor is shown in a scatter plot. TISIDB (http://cis.hku.hk/TISIDB/: accessed on 13 May 2023), a web tool for investigating tumor and immune system interactions [91], was also used to determine the correlation between the *PKHD1L1* expression and 28 types of TILs. The correlation coefficient between the expression levels of *PKHD1L1* and TILs was determined using the Spearman’s test. The correlation between the expression levels of *PKHD1L1* with the genetic signatures of B cells, cluster of differentiation (CD)-8^+^ T cells, and NK cells was analyzed using Spearman’s correlation test in the TIMER v.2.0 database (http://timer.cistrome.org/: accessed on 3 May 2023) [92]. We also compared the infiltration levels of activated and resting NK cells using CIBERSORT NK signatures in TIMER v.2.0.

### 4.5. Identification of Co-Expressed Genes with PKHD1L1

The co-expressed gene profile of *PKHD1L1* was analyzed using the TCGA-SKCM and TCGA-LUAD datasets with the cBioportal database (https://www.cbioportal.org/: accessed on 23 May 2023) [93]. We used the TCGA database in the UCSC Xena browser to visualize the correlation between the most highly positively correlated genes and *PKHD1L1* using heatmaps and dot plots (http://xena.ucsc.edu/: accessed on 17 July 2023) [94]. Scatter plots of the expressions of *PKHD1L1* and the most highly positively correlated genes were identified in other lung and skin cancer datasets using R2: Genomics Analysis and Visualization Platform (https://hgserver1.amc.nl/cgi-bin/r2/main.cgi: accessed on 28 July 2023).

### 4.6. Cluster Analysis of PKHD1L1 and Its Co-Expressed Genes

The GeneMANIA web tool (http://www.enenmania.org/: accessed on 13 August 2023) was used for the protein–protein interaction analysis [95]. We used this database to predict the protein–protein interactions using *PKHD1L1* as the query. A Venn diagram was used to identify the common co-expressed genes in SKCM and LUAD. We analyzed the ontology of *PKHD1L1* and the common co-expressed genes in SKCM and LUAD using the Enrichr web tool (https://amp.pharm.mssm.edu/Enrichr: accessed on 15 August 2023) [96]. Gene ontology (GO) and pathway analyses were performed using bar diagrams.

### 4.7. Statistical Analysis

All data were analyzed for the *p*-values and number of samples using various standard statistical methods in various databases. The detailed statistical tests are presented in Supplementary Table S2.

## 5. Conclusions

In conclusion, this study showed the downregulation of the *PKHD1L1* mRNA expression in SKCM and LUAD compared to normal tissues. Moreover, the *PKHD1L1* expression was strongly correlated with a good prognosis and infiltration levels of immune cells, including B, CD8^+^ T, and NK cells. Therefore, our systematic analysis suggests the clinical relevance and prognostic value of the expression levels of *PKHD1L1* in SKCM and LUAD.

## Figures and Tables

**Table 1:** Infiltration levels of *PKHD1L1* using the Tumor Immune Estimation Resource (TIMER), v.1.0.

Cancer	SKCM		LUAD	
	Correlation	*p*	Correlation	*p*
Purity	−0.40445	1.88 × 10^−19^	−0.35726	2.56 × 10^−16^
B cells	0.370997	4.58 × 10^−16^	0.419973	4.2 × 10^−22^
CD8^+^ T cells	0.390797	1.97 × 10^−17^	0.335172	3 × 10^−14^
CD4^+^ T cells	0.354657	1.15 × 10^−14^	0.199543	9.73 × 10^−6^
Macrophages	0.21392	4.35 × 10^−6^	0.196683	1.28 × 10^−5^
Neutrophils	0.409228	1.12 × 10^−19^	0.252514	1.84 × 10^−8^
Dendritic Cells	0.34351	8.49 × 10^−14^	0.208905	3.25 × 10^−6^

SKCM, skin cutaneous melanoma; LUAD, lung adenocarcinoma.

**Table 2:** Correlation between *PKHD1L1* expression and immune cell infiltration using TIMER 2.0.

Immune Cells	Statistical Test Method	SKCM (*n* = 471)		LUAD (*n* = 515)	
		Rho	*p*	Rho	*p*
B cells	EPIC	0.512	5.83 × 10^−32^	0.519	2.32 × 10^−35^
	QUANTISEQ	0.531	1.30 × 10^−34^	0.497	4.44 × 10^−32^
	MCP_COUNTER	0.542	2.56 × 10^−36^	0.551	2.08 × 10^−40^
	CIBERSORT_ABS	0.403	2.53 × 10^−12^	0.403	1.01 × 10^−20^
CD8^+^ T cells	MCP_COUNTER	0.433	7.25 × 10^−72^	0.279	2.73 × 10^−10^
	CIBERSORT_ABS	0.436	3.49 × 10^−50^	0.365	3.49 × 10^−50^
	QUANTISEQ	0.36	6.91 × 10^−14^	0.287	6.91 × 10^−14^
	XCELL	0.347	6.91 × 10^−14^	0.259	6.91 × 10^−14^
NK cells	MCP_COUNTER	0.433	2.40 × 10^−22^	0.263	1.47 × 10^−10^
	Activated CIBERSORT_ABS	0.326	9.09 × 10^−13^	0.22	8.399 × 10^−7^
	Resting CIBERSORT	−0.196	2.50 × 10^−5^	−0.196	2.16 × 10^−1^
	Resting CIBERSORT_ABS	−0.164	4.45 × 10^−4^	−0.05	2.68 × 10^−1^

SKCM, skin cutaneous melanoma; LUAD, lung adenocarcinoma; NK, natural killer. Positive correlation (*p* < 0.05, rho > 0), negative correlation (*p* < 0.05, rho < 0), not significant (*p* > 0.05).

## Data Availability

The datasets presented in this study can be found in online repositories. The names of the repositories and accession number(s) can be found in the article/Supplementary Materials.

## Supplementary Materials

The following supporting information can be downloaded at https://www.mdpi.com/article/10.3390/ijms25010359/s1.
