# Supplementary material for: Systematic Multiomic Analysis of PKHD1L1 Gene Expression and Its Role as a Predicting Biomarker for Immune Cell Infiltration in Skin Cutaneous Melanoma and Lung Adenocarcinoma
Source: Int J Mol Sci. 2023 Dec 26;25(1):359. doi: 10.3390/ijms25010359 (PMC10778817; doi:10.3390/ijms25010359)
Supplement: Supplementary file 1 [file ijms-25-00359-s001.zip › ijms-2734654-supplementary/Supplementary Figure S1.PDF]

Supplementary Figure S1. Overall survival between PKHD1L1 expression and the two patient groups in skin cutaneous melanoma (SKCM) and lung adenocarcinoma (LUAD) with gender, age, and tumor stage. Kaplan-meier survival curves of high and low patient groups the split by PKHD1L1 expression with the scan-cut-off modus were scanned by R2 database with the SKCM-TCGA and LUAD-TCGA dataset. (*p < 0.05)

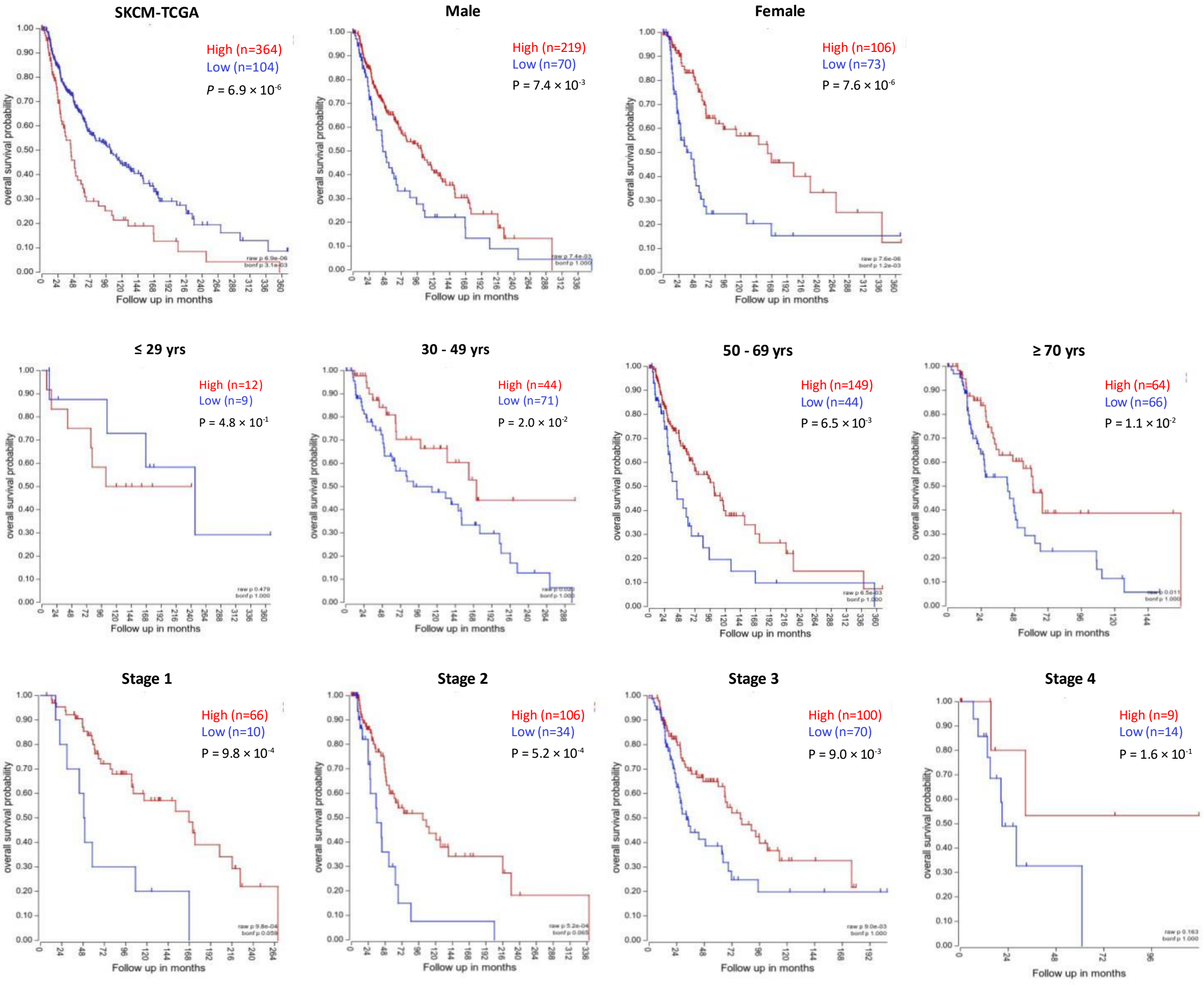

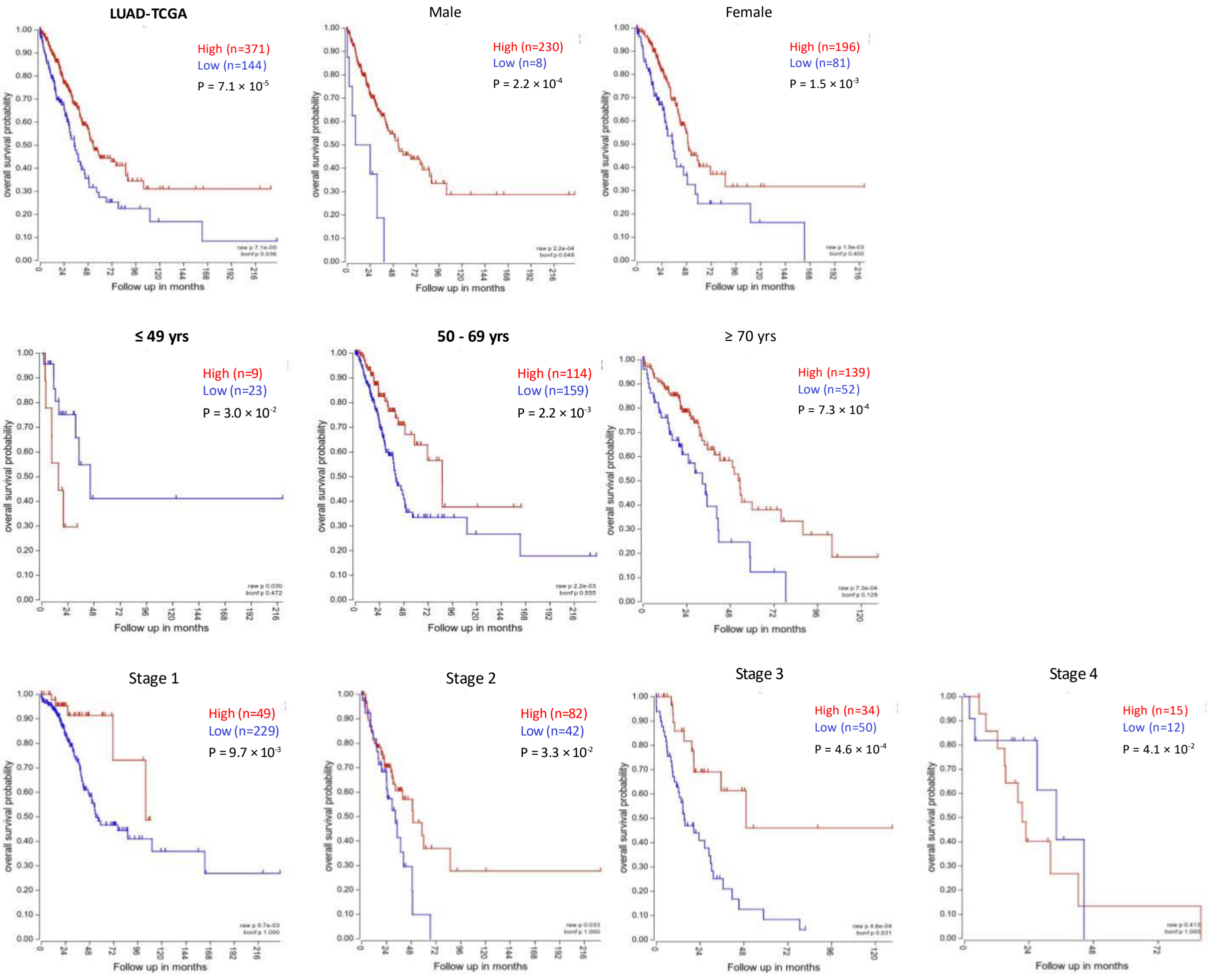

LUAD-TCGA
High (n=371)
Low (n=144)
P = 7.1 × 10-5
Male
High (n=230)
Low (n=8)
P = 2.2 × 10-4
Female
High (n=196)
Low (n=81)
P = 1.5 × 10-3
≤ 49 yrs
High (n=9)
Low (n=23)
P = 3.0 × 10-2
50 - 69 yrs
High (n=114)
Low (n=159)
P = 2.2 × 10-3
≥ 70 yrs
High (n=139)
Low (n=52)
P = 7.3 × 10-4
Stage 1
High (n=49)
Low (n=229)
P = 9.7 × 10-3
Stage 2
High (n=82)
Low (n=42)
P = 3.3 × 10-2
Stage 3
High (n=34)
Low (n=50)
P = 4.6 × 10-4
Stage 4
High (n=15)
Low (n=12)
P = 4.1 × 10-2
overall survival probability
Follow up in months
