# Supplementary material for: Systematic Multiomic Analysis of PKHD1L1 Gene Expression and Its Role as a Predicting Biomarker for Immune Cell Infiltration in Skin Cutaneous Melanoma and Lung Adenocarcinoma
Source: Int J Mol Sci. 2023 Dec 26;25(1):359. doi: 10.3390/ijms25010359 (PMC10778817; doi:10.3390/ijms25010359)

Supplementary Figure S2. Correlation between PKHD1L1 expression levels and patient survival rates in the kidney renal papillary cell carcinoma (KIRP), pancreatic adenocarcinoma (PAAD), and stomach adenocarcinoma (STAD) using the Gene Expression Profiling Interactive Analysis 2 (GEPIA2) survival curve. (*p < 0.05)

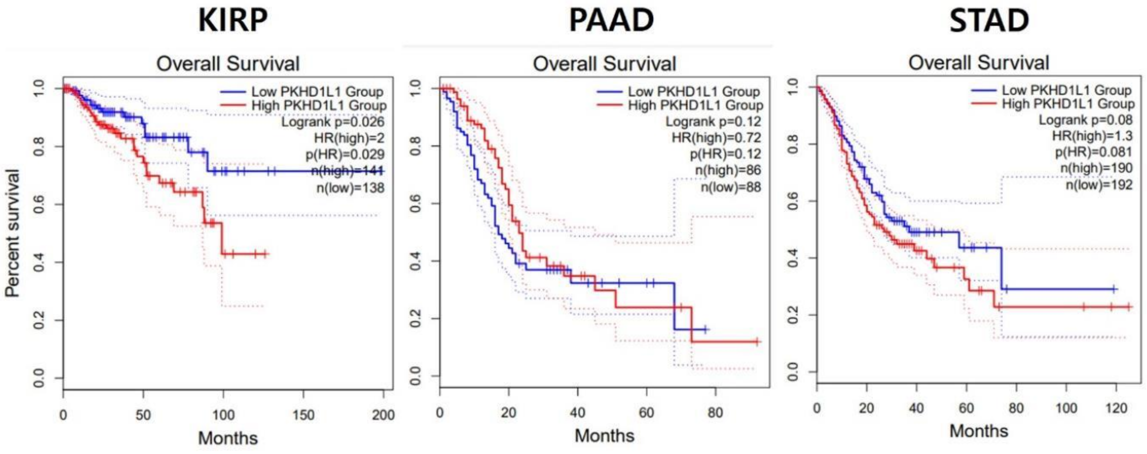

Supplement: Supplementary file 1 [file ijms-25-00359-s001.zip › ijms-2734654-supplementary/Supplementary Figure S2.PDF]
