# Supplementary material for: Systematic Multiomic Analysis of PKHD1L1 Gene Expression and Its Role as a Predicting Biomarker for Immune Cell Infiltration in Skin Cutaneous Melanoma and Lung Adenocarcinoma
Source: Int J Mol Sci. 2023 Dec 26;25(1):359. doi: 10.3390/ijms25010359 (PMC10778817; doi:10.3390/ijms25010359)

Supplementary Figure S3. Correlations between the expression levels of *PKHD1L1* and lymphocytes. (**A**) *PKHD1L1* expression levels in common immune cell types were retrieved from RNA-seq-based expression quantitative trait loci (eQTLs) and epigenomics (DICE) database (https://dice-database.org/landing). *PKHD1L1* expression levels (x-axis) were represented as the number of transcripts per million transcripts (TPM). Common immune cell types (y-axis) are arranged based on the median expression level within the cohort from highest to lowest. Box plots indicate 25-75% ranges, and whiskers indicate minimum to maximum. (**B**) Common immune cell types (y-axis) are separated in detail by female–male. (**C**) Single cell analysis of melanoma intra-tumor heterogeneity, using the Single Cell Portal (https://singlecell.broadinstitute.org/single_cell), enriched for population cell subtypes, represented as violin plots. (**D**) t-Distributed Stochastic Neighbor Embedding (tSNE) visualization of immune cell populations identified through unsupervised cluster analysis of *PKHD1L1* expression from melanoma intra-tumor heterogeneity. Seven clusters including B cell (red), CAF (light blue), endothelial cluster (green), macrophage (purple), NK cell (orange), T cell (brown), and undefined (pink) were colorfully visualized.

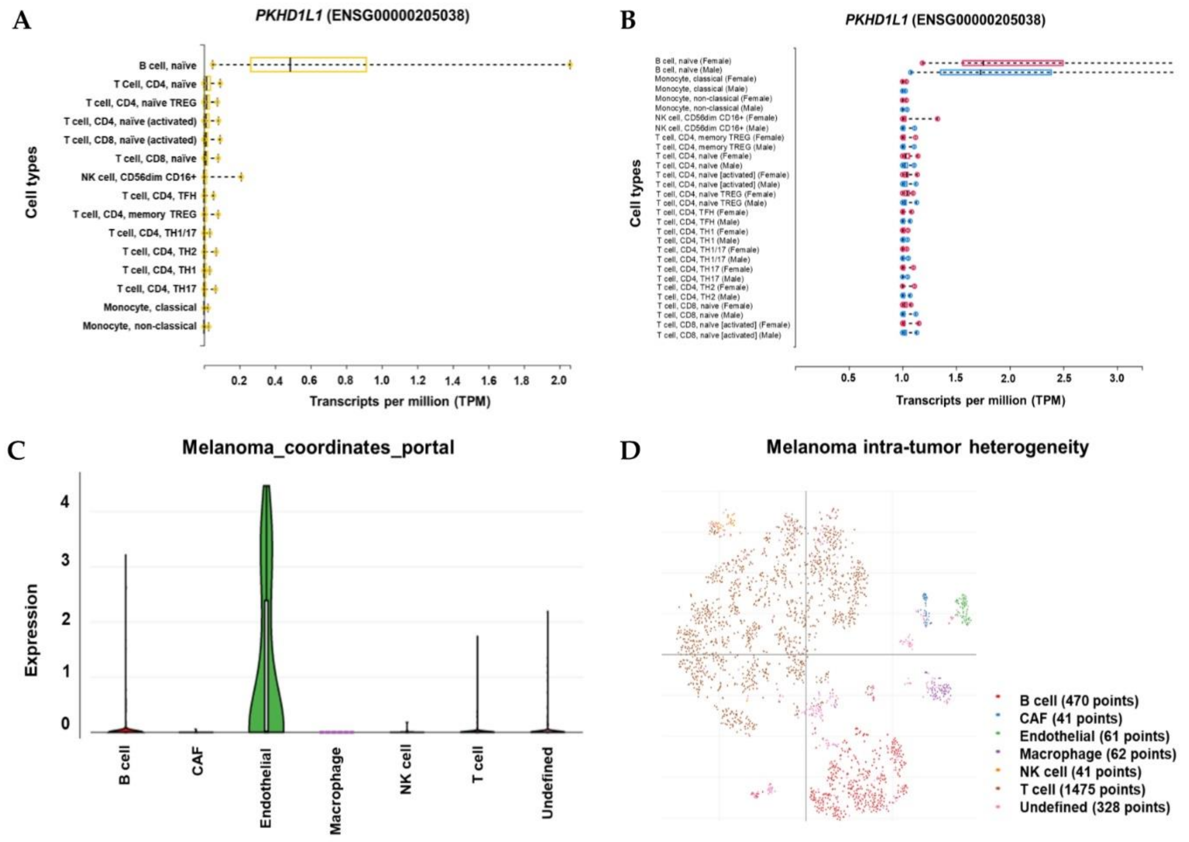

Supplement: Supplementary file 1 [file ijms-25-00359-s001.zip › ijms-2734654-supplementary/Supplementary Figure S3.PDF]
