# Supplementary material for: Systematic Multiomic Analysis of PKHD1L1 Gene Expression and Its Role as a Predicting Biomarker for Immune Cell Infiltration in Skin Cutaneous Melanoma and Lung Adenocarcinoma
Source: Int J Mol Sci. 2023 Dec 26;25(1):359. doi: 10.3390/ijms25010359 (PMC10778817; doi:10.3390/ijms25010359)

Supplementary Figure S4. Correlation of chemokine expression with PKHD1L1 expression in various cancers. (A) Spearman correlations between PKHD1L1 expression and chemokines (Y axis) across human cancers (X axis) using TISIDB. The value in the following heatmap represents rho value (-1 : blue ~1 : red). (B) Association between 4 chemokines expression and PKHD1L1 expression in SKCM (n=472) and LUAD (n=517) analyzed by TISIDB. 4 chemokines (CCL4, CCL5, CCL19, CXCL9) show positive correlation with PKDH1L1 in SKCM and LUAD.

A

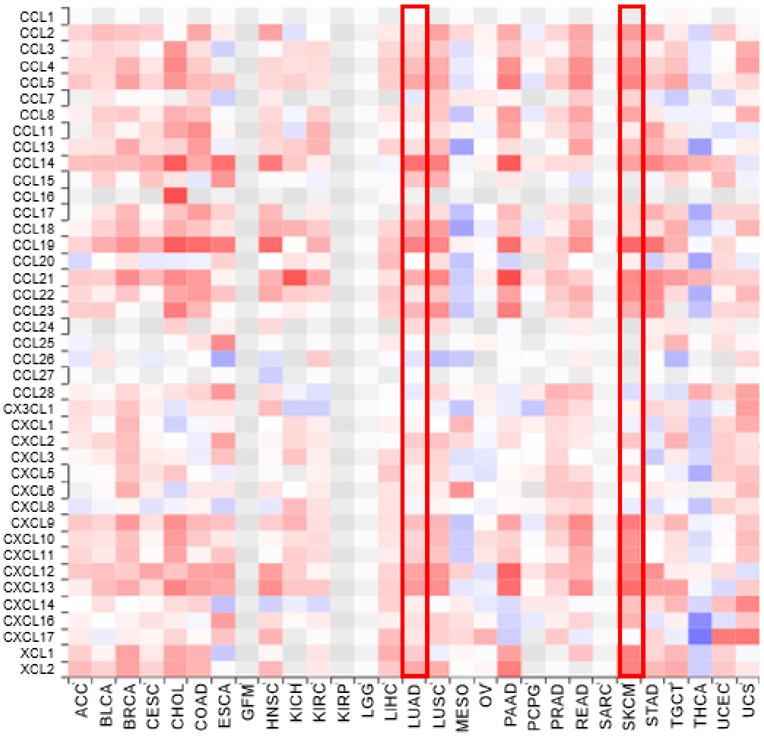

B

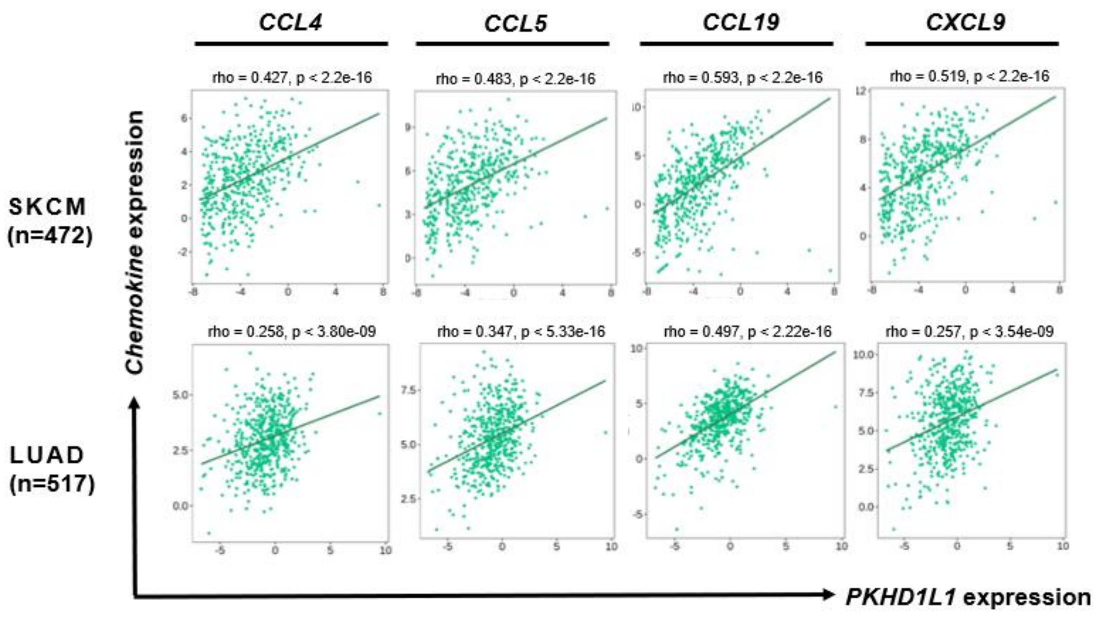

Supplement: Supplementary file 1 [file ijms-25-00359-s001.zip › ijms-2734654-supplementary/Supplementary Figure S4.PDF]
