# Supplementary material for: Systematic Multiomic Analysis of PKHD1L1 Gene Expression and Its Role as a Predicting Biomarker for Immune Cell Infiltration in Skin Cutaneous Melanoma and Lung Adenocarcinoma
Source: Int J Mol Sci. 2023 Dec 26;25(1):359. doi: 10.3390/ijms25010359 (PMC10778817; doi:10.3390/ijms25010359)
Supplement: Supplementary file 1 [file ijms-25-00359-s001.zip › ijms-2734654-supplementary/Supplementary Table S2.PDF]

Supplementary Table S2. Statistical tests used in Figures data.

Figure	Database	Object	Method	Sample		Statistical test	p-value ($p<0.05$)
**Figure 1**	GEPIA2	mRNA Expression level, Box plot	TCGA & GTEx projects	SKCM	T(n=461)<br>N(n=558)	ANOVA<br>Empirical Bayes method<br>One-way ANOVA	$p=2.94\times10^{-111}$<br>(Log2FC=-1.521)
				LUAD	T(n=483)<br>N(n=347)	ANOVA<br>Empirical Bayes method<br>One-way ANOVA	$p=1.66\times10^{-117}$<br>(Log2FC=-2.379)
	GENT2	Gene expression	Microarray datasets	SKCM	T(n=547)<br>N(n=263)	Two-sample T-test	$p<0.0001$<br>(Log2FC= -1.136)
				LUAD	T(n=2362)<br>N(n=508)		$p<0.0001$<br>(Log2FC= -0.767)
**Figure 2**	GEPIA2	Survival plot (Overall)	TCGA & GTEx projects	SKCM	High(n=223)<br>Low(n=221)	Log rank test (Mantel-Cox test)	$p=1.8\times10^{-6}$
				LUAD	High(n=239)<br>Low(n=238)		$p=1.4\times10^{-3}$
	Prognoscan	Survival plot (OS, RFS)	Microarray datasets	SKCM	High(n=11)<br>Low(n=27)	Minimum P-value approach	$p=4.23\times10^{-2}$
				LUAD	High(n=117)<br>Low(n=21)		$p=3.41\times10^{-2}$
**Figure 4**	TISIDB	Expression of immune cells	gene set variation analysis (GSVA)	SKCM	n=472	Empirical bayes estimate	Activated B cell ($p<2.2\times10^{-16}$)<br>Activated CD8+ T cell ($p<2.2\times10^{-16}$)<br>Effector memory CD8+ T cell ($p<2.2\times10^{-16}$)<br>NK cell ($p<2.2\times10^{-16}$)
				LUAD	n=517		Activated B cell ($p<2.2\times10^{-16}$)<br>Activated CD8+ T cell ($p<1.84\times10^{-11}$)<br>Effector memory CD8+ T cell ($p<2.15\times10^{-16}$)<br>NK cell ($p<1.61\times10^{-11}$)

**Figure 5**	TIMER Version 2.0	Correlation	EPIC<br>QUANTISEQ<br>MCP_COUNTER<br>CIBERSORT_ABS	SKCM (n=471)<br>LUAD (n=515)	Bayesian statistics<br>Gibbs sampling<br>Expectation maximization algorithm	B cell (SKCM)	($p=5.83\times10^{-32}$)<br>($p=1.30\times10^{-34}$)<br>($p=2.56\times10^{-36}$)<br>($p=2.53\times10^{-12}$)
						B cell (LUAD)	($p=2.32\times10^{-35}$)<br>($p=4.44\times10^{-32}$)<br>($p=2.08\times10^{-40}$)<br>($p=1.01\times10^{-20}$)
			MCP_COUNTER<br>CIBERSORT-ABS<br>QUANTISEQ<br>XCELL		Bayesian statistics<br>Gibbs sampling<br>Expectation maximization algorithm	CD+8 T cell (SKCM)	($p=7.25\times10^{-72}$)<br>($p=3.49\times10^{-50}$)<br>($p=6.91\times10^{-14}$)<br>($p=6.91\times10^{-14}$)
						CD+8 T cell (LUAD)	($p=2.73\times10^{-10}$)<br>($p=3.49\times10^{-50}$)<br>($p=6.91\times10^{-14}$)<br>($p=6.91\times10^{-14}$)
			MCP_COUNTER<br>Activated CIBERSORT-ABS<br>Resting CIBERSORT<br>Resting CIBERSORT-ABS		Bayesian statistics<br>Gibbs sampling<br>Expectation maximization algorithm	NK cell (SKCM)	($p=2.40\times10^{-22}$)<br>($p=9.09\times10^{-13}$)<br>($p=2.50\times10^{-5}$)<br>($p=4.45\times10^{-4}$)
						NK cell (LUAD)	($p=1.47\times10^{-10}$)<br>($p=8.399\times10^{-7}$)<br>($p=2.16\times10^{-1}$)<br>($p=2.68\times10^{-1}$)
**Figure 6**	UCSC Xena	Profiling of co-expressed genes with PKHD1L1	TCGA	SKCM(n=481)			$p=2.53\times10^{-81}$
				LUAD(n=706)			$p=3.228\times10^{-35}$
	R2 platform		TCGA	SKCM(n=80)			$p=1.45\times10^{-4}$
				LUAD(n=410)			$p=5.97\times10^{-64}$
