# Supplementary material for: Systematic Multiomic Analysis of PKHD1L1 Gene Expression and Its Role as a Predicting Biomarker for Immune Cell Infiltration in Skin Cutaneous Melanoma and Lung Adenocarcinoma
Source: Int J Mol Sci. 2023 Dec 26;25(1):359. doi: 10.3390/ijms25010359 (PMC10778817; doi:10.3390/ijms25010359)
Supplement: Supplementary file 1 [file ijms-25-00359-s001.zip › ijms-2734654-supplementary/Supplementary Table S3.pdf]

Supplementary Table S3. Cox regression result for polycystic kidney and hepatic disease 1-like protein 1 (*PKHD1L1*) with The Cancer Genome Atlas (TCGA) data in various types of cancers using the OncoLnc database. (*$p < 0.01$)

Cancer	Cox coefficient	*P*-Value	FDR corrected	Rank	Median expression	Mean expression
LUAD	- 0.293	9.80×10^{-5}	8.74×10^{-3}	185	12.2	49.94
SKCM	- 0.237	2.20×10^{-3}	2.41×10^{-2}	1455	1.09	17.07
KIRP	0.412	9.40×10^{-3}	4.75×10^{-2}	3255	2.11	3.81
PAAD	- 0.252	4.20×10^{-2}	1.65×10^{-1}	4358	3.79	12.55
STAD	0.169	4.20×10^{-2}	3.23×10^{-1}	2195	6.81	26.39
HNSC	- 0.116	1.20×10^{-1}	4.43×10^{-1}	4432	2.16	7.18
BLCA	- 0.114	1.60×10^{-1}	4.06×10^{-1}	6390	1.43	5.37
BRCA	- 0.129	1.60×10^{-1}	5.32×10^{-1}	4937	2.91	8.35
ESCA	- 0.21	1.60×10^{-1}	9.53×10^{-1}	2751	2.33	10.41
LUSC	0.085	2.50×10^{-1}	7.44×10^{-1}	5649	7.3	17.89
LIHC	0.108	2.70×10^{-1}	5.34×10^{-1}	7970	2.34	11.67
COAD	- 0.102	3.50×10^{-1}	7.29×10^{-1}	7831	2.46	5.23
KIRC	0.074	3.50×10^{-1}	4.81×10^{-1}	12105	1.56	4.73
READ	- 0.188	4.50×10^{-1}	9.62×10^{-1}	7662	2.28	3.53
UCEC	- 0.075	4.90×10^{-1}	9.95×10^{-1}	8144	196.45	1591.71
CESC	0.045	7.30×10^{-1}	9.02×10^{-1}	13204	2.23	59.04
OV	- 0.024	7.60×10^{-1}	9.59×10^{-1}	13358	106.65	675.66
LAML	- 0.001	9.90×10^{-1}	9.95×10^{-1}	15159	5.49	22.45
