# Supplementary material for: Systematic Multiomic Analysis of PKHD1L1 Gene Expression and Its Role as a Predicting Biomarker for Immune Cell Infiltration in Skin Cutaneous Melanoma and Lung Adenocarcinoma
Source: Int J Mol Sci. 2023 Dec 26;25(1):359. doi: 10.3390/ijms25010359 (PMC10778817; doi:10.3390/ijms25010359)
Supplement: Supplementary file 1 [file ijms-25-00359-s001.zip › ijms-2734654-supplementary/Supplementary Table S4.PDF]

Supplementary Table S4. Co-expressed genes in SKCM and LUAD in Figure 6

**1**	CASS4	**41**	ARHGAP30	**81**	GMFG	**121**	IL7R	**161**	NCF1	**201**	RGL4
**2**	RBP5	**42**	TLR10	**82**	TMEM273	**122**	MAP4K1	**162**	CD2	**202**	ITGAL
**3**	GCSAM	**43**	JAML	**83**	SLAMF1	**123**	ZNF80	**163**	IRF8	**203**	THEMIS
**4**	LGALS2	**44**	SEPTIN1	**84**	RASGRP2	**124**	FMO2	**164**	CXCR6	**204**	PTPN7
**5**	ZAP70	**45**	PTGDR	**85**	FCRL3	**125**	TBX21	**165**	HLA-DOB	**205**	IL18RAP
**6**	BTG2	**46**	PIK3CG	**86**	IKZF3	**126**	IL10RA	**166**	CD200R1	**206**	JCHAIN
**7**	PTPRCAP	**47**	NIBAN3	**87**	ELMO1	**127**	GAB3	**167**	ABI3BP	**207**	CSF2RB
**8**	SPIB	**48**	CCL19	**88**	RGS13	**128**	TNFRSF13B	**168**	WDFY4	**208**	SLAMF6
**9**	SLA2	**49**	TESPA1	**89**	CYTIP	**129**	KLHL6	**169**	LY9	**209**	PTGER4
**10**	CXCR4	**50**	IKZF1	**90**	CLEC17A	**130**	CD96	**170**	KCNN3	**210**	CCR7
**11**	FGL2	**51**	TCL1A	**91**	SCIMP	**131**	CCDC69	**171**	BMS1P20	**211**	CR1
**12**	CRTAM	**52**	ZC3H12D	**92**	ADGRG5	**132**	CR2	**172**	FLI1	**212**	PRDM1
**13**	CD37	**53**	CD79A	**93**	GVINP1	**133**	LCK	**173**	ANKRD36BP2	**213**	KLRB1
**14**	NCF1C	**54**	S1PR1	**94**	CD53	**134**	TLR9	**174**	MFNG	**214**	NLRC3
**15**	NCF1B	**55**	GIMAP7	**95**	RASGRP3	**135**	TIFAB	**175**	ITK	**215**	FGD2
**16**	PARP15	**56**	GIMAP1	**96**	CD247	**136**	KLRK1	**176**	CD69	**216**	P2RY14
**17**	BTLA	**57**	FLT3	**97**	TENT5C	**137**	PPP1R16B	**177**	LTA	**217**	UBASH3A
**18**	GPR65	**58**	DOCK8	**98**	ITGA8	**138**	NCR3	**178**	SIT1	**218**	ZNF831
**19**	KBTBD8	**59**	PLA2G2D	**99**	PNOC	**139**	CCR2	**179**	FAM30A	**219**	ACAP1
**20**	PECAM1	**60**	POU2AF1	**100**	LRMP	**140**	TRAF3IP3	**180**	GGTA1P	**220**	PTPRC
**21**	MS4A1	**61**	MZB1	**101**	ARHGAP15	**141**	NUGGC	**181**	GIMAP6	**221**	CCR4
**22**	ADA2	**62**	GIMAP4	**102**	P2RY10	**142**	PRKCQ	**182**	VNN2	**222**	IL2RG
**23**	ADAM6	**63**	CD28	**103**	FCRL4	**143**	FCER2	**183**	CLEC10A	**223**	CXCR5
**24**	DOCK2	**64**	SPOCK2	**104**	INMT	**144**	MEI1	**184**	TSHR		
**25**	BTN1A1	**65**	TBC1D10C	**105**	P2RX1	**145**	P2RY8	**185**	ART4		
**26**	BANK1	**66**	SASH3	**106**	CD48	**146**	DCANP1	**186**	GPR18		
**27**	SPN	**67**	BIN2	**107**	GZMK	**147**	CD27	**187**	AOAH		
**28**	CD1D	**68**	AICDA	**108**	ITM2A	**148**	TNFRSF17	**188**	ZBP1		
**29**	CNR2	**69**	CD6	**109**	VSIR	**149**	CD226	**189**	TNFRSF13C		
**30**	CD244	**70**	SP140	**110**	FCRL5	**150**	S1PR4	**190**	PCED1B-AS1		
**31**	SH2D1A	**71**	EVI2B	**111**	TRAT1	**151**	MPEG1	**191**	ZBTB32		
**32**	GIMAP8	**72**	TNFSF8	**112**	XCR1	**152**	CD8A	**192**	CD3E		
**33**	PLCG2	**73**	SLC9A9	**113**	STAP1	**153**	FGD3	**193**	PAX5		
**34**	PLEK	**74**	CD52	**114**	ABCD2	**154**	C5ORF56	**194**	FCRL1		

**35**	ARHGAP9	**75**	NCKAP1L	**115**	CD5	**155**	ARHGAP25	**195**	POU2F2
**36**	SAMD3	**76**	ADORA2A	**116**	CD3G	**156**	AMPD1	**196**	BLK
**37**	LAX1	**77**	RCSD1	**117**	BTK	**157**	GIMAP5	**197**	BEND4
**38**	C16ORF54	**78**	RHOH	**118**	CD180	**158**	GPR174	**198**	PIM2
**39**	PRKCB	**79**	VPREB3	**119**	CXORF21	**159**	FCRL2	**199**	CD40LG
**40**	PIK3R5	**80**	CD19	**120**	TAGAP	**160**	RASAL3	**200**	AGAP2
